# A five‐microRNA signature for individualized prognosis evaluation and radiotherapy guidance in patients with diffuse lower‐grade glioma

**DOI:** 10.1111/jcmm.15377

**Published:** 2020-05-15

**Authors:** Jian‐Hua Zhang, Ruiqin Hou, Yuhualei Pan, Yuhan Gao, Ying Yang, Wenqin Tian, Yan‐Bing Zhu

**Affiliations:** ^1^ Department of Blood Transfusion Peking University People's Hospital Beijing China; ^2^ Experimental and Translational Research Center Beijing Friendship Hospital Affiliated to the Capital University of Medical Sciences Beijing China

**Keywords:** diffuse lower‐grade glioma, microRNA, prognostic, radiotherapeutic response, signature

## Abstract

Despite the prognostic value of IDH and other gene mutations found in diffuse glioma, markers that judge individual prognosis of patients with diffuse lower‐grade glioma (LGG) are still lacking. This study aims to develop an expression‐based microRNA signature to provide survival and radiotherapeutic response prediction for LGG patients. MicroRNA expression profiles and relevant clinical information of LGG patients were downloaded from The Cancer Genome Atlas (TCGA; the training group) and the Chinese Glioma Genome Atlas (CGGA; the test group). Cox regression analysis, random survival forests‐variable hunting (RSFVH) screening and receiver operating characteristic (ROC) were used to identify the prognostic microRNA signature. ROC and TimeROC curves were plotted to compare the predictive ability of IDH mutation and the signature. Stratification analysis was conducted in patients with radiotherapy information. Gene ontology (GO) analysis and Kyoto Encyclopedia of Genes and Genomes (KEGG) pathway analysis were performed to explore the biological function of the signature. We identified a five‐microRNA signature that can classify patients into low‐risk or high‐risk group with significantly different survival in the training and test datasets (*P* < 0.001). The five‐microRNA signature was proved to be superior to IDH mutation in survival prediction (AUCtraining = 0.688 vs 0.607). Stratification analysis found the signature could further divide patients after radiotherapy into two risk groups. GO and KEGG analyses revealed that microRNAs from the prognostic signature were mainly enriched in cancer‐associated pathways. The newly discovered five‐microRNA signature could predict survival and radiotherapeutic response of LGG patients based on individual microRNA expression.

## 1 INTRODUCTION

Glioma is a common type of intracranial malignant tumour, characterized by diffuse infiltration, no clear boundary, infinite proliferation and high invasion. According to histomorphological classification, World Health Organization (WHO) classifies gliomas into grades I to IV for diagnosis, guidance treatment and prognosis. Grade I gliomas have the lowest invasiveness and the best prognosis. Generally, patients can be cured by surgical removal of tumour tissue, and the 5‐year survival rate reaches 94%.^1^ On the contrary, grade IV tumours, in which glioblastomas are the majority, are the most aggressive and malignant. Even after maximal resection plus post‐operative chemoradiotherapy, the median survival of patients with glioblastoma is as short as about one year.^2^ Gliomas of grades II and III are intermediate in terms of malignant degree and prognosis and are combined into lower‐grade gliomas because of their similarity in high invasiveness and the tendency to recur or develop advanced lesions such as secondary glioblastoma. Especially, the grade II/III astrocytic tumours and the grade II/III oligodendrogliomas (oligoastrocytomas are sorted into either astrocytoma or oligodendroglioma) are incorporated into diffuse lower‐grade gliomas (LGG) after the 2016 CNS WHO Classification.^3^ Some studies have found little prognostic difference between grade II diffuse astrocytomas and grade III anaplastic astrocytomas.^4^, ^5^ However, not all studies have confirmed the prognostic consistency of grade II and grade III gliomas.^6^ Variabilities responding to treatment and differences in the timing of disease progression or recurrence are also frequently seen in LGG. Therefore, for patients with LGG, prognostic markers that can predict individual clinical outcome and treatment response are essential.

With the introduction of IDH mutation and 1p/19g codeletion into the classification of diffuse gliomas, it is increasingly recognized that molecular features play a significant role in the evaluation of prognosis and treatment response. MicroRNAs are a group of short‐chain non‐coding RNA molecules that are involved in the regulation multiple genes expression and activation of signalling pathways. Abnormal microRNA expression occurs in a variety of tumours and is closely related to tumour invasion, metastasis and more malignant lesions. Thus, microRNA could be used to predict treatment response and prognosis of many types of cancer and become a molecular marker. In addition, the stability and detectability of microRNAs in a variety of body fluids (serum/plasma/cerebrospinal fluid) are more favourable for their use as prognostic markers. Therefore, microRNA signature containing multiple microRNAs has been demonstrated its promising prognostic role in various tumours including breast cancer,^7^ hepatocellular carcinoma^8^ and glioblastoma.^9^ However, there is a lack of microRNA signature that can predict individual outcomes of patients with LGG.

In the present study, LGG patients with microRNA expression data were collected from the Cancer Genome Atlas (TCGA) database and the Chinese Glioma Genome Atlas (CGGA). We aim to use these expression data and clinical data to identify a prognostic microRNA signature and validate its predictive ability for survival and treatment response.

## 2 MATERIALS AND METHODS

### 2.1 Diffuse lower‐grade glioma patient cohorts and data collection

One dataset with 530 LGG cases and microRNA expression data were collected from the TCGA (http://cancergenome.nih.gov/, https://xenabrowser.net/datapages/), of which 525 LGG patients had survival time and survival status. Another dataset with 107 LGG cases was downloaded from CGGA database (http://www.cgga.org.cn/), and we found 99 cases with survival information. Then, the 525 LGG cases from TCGA and 99 LGG cases from CGGA were treated as the training and test datasets, respectively. The clinical information of all 624 patients was shown in Table 1. There were 1881 microRNAs involved for further analysis, and log2 transformation was performed for the original expression value of microRNAs.

**TABLE 1:** Summary of the patient demographics and clinical characteristics

Characteristic	Training dataset (n = 525)	Test dataset (n = 99)
Age (y)		
>40	264	41
≤40	261	57
Unknown		1
Sex		
Female	239	46
Male	286	53
Vital status		
Living	389	49
Dead	136	50
Grade		
G2	255	55
G3	269	44
Unknown	1	
IDH mutation status		
Mutant	88	64
Wild‐type	34	30
Unknown	403	5
Chemo status		
No		44
Yes		53
Unknown	525	2
Radio status		
No	174	9
Yes	285	89
Unknown	66	1

### 2.2 Developing prognostic microRNA signatures in the TCGA dataset

Through univariate COX proportional regression analysis, we first identified the microRNAs associated with patients' survival time from the training group. Then, we further reduced the number of the survival related microRNAs using the random survival forests‐variable hunting (RSFVH) algorithm. In RSFVH, an iteration procedure was performed to narrow down the microRNAs set in which the 1/4 least important microRNAs were discarded at each iteration step and five hundred trees were grown at each step until without changing the error rate too much and then 9 microRNAs were selected.^10^, ^11^ Finally, COX regression analysis of selected candidate microRNAs was performed and prognostic models were constructed as follows: $\text{microRNA Risk Score}=\sum_{i=1}^{N}\left({\text{Expression}}_{i}\times \beta_{i}\right)$ , where N is the number of microRNA, expression is the microRNA expression value and *β* is the microRNA estimated regression coefficient in the Cox regression analysis.^12^, ^13^ The final prognostic microRNA signature was screened out by comparing area under the ROC curve (AUC) values of prognostic models.^14^

### 2.3 Statistical and bioinformatics analysis

The median risk score was used as the cut‐off value to subdivide patients with LGG into two risk groups. Kaplan‐Meier analysis was performed to assess the survival difference of two risk groups, and statistical significance was assessed using the two‐sided log‐rank test. Multivariate Cox regression analysis was performed to explore the independence of the screened signature. ROC and TimeROC were used to compare predictive performance. All analyses were performed with R program (www.r‐project.org), including packages named pROC, TimeROC, randomForestSRC and survival downloaded from Bioconductor (http://www.bioconductor.org/). MicroRNA function was analysed by DIANA‐mirPath, which is a web‐based computational tool developed to identify molecular pathways potentially for single or multiple microRNAs by performing an enrichment analysis of multiple microRNA target genes.^15^, ^16^

## 3 RESULTS

### 3.1 Identification of prognostic microRNAs and construction of microRNA signature in diffuse lower‐grade glioma

A total of 624 LGG patients with their microRNA expression data and clinical follow‐up information were collected from the TCGA and CGGA database. Based on statistical analysis of the clinical data, we found that the median age of the enrolled patients was 40 years (14‐87 years) and male patients were more than female ones. The number of grade II cases was basically the same as that of grade III, accounting for roughly half. Every patient had survival information, all but 186 of whom died. In addition, we listed the histological type, IDH mutation and radiotherapy and chemotherapy information (Table 1).

**FIGURE 1:**
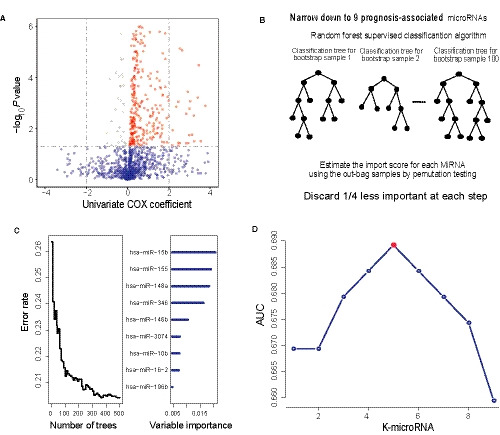
Identification of the prognostic signature in the training dataset. (A) Volcano plot displayed the survival associated microRNAs in univariate cox regression analysis. Grey dots were protective microRNAs with negative coefficient, and red dots were risking microRNAs with positive coefficient (B, C) Random forest supervised classification algorithm reduced the prognosis‐associated microRNAs to 9 microRNAs.(D) After calculating the AUC of 2^9^ − 1 = 511 signatures, the prognostic five‐microRNA signature with the largest prediction power (AUC = 0.69) was screen out

Using microRNA profiling data and survival information from the training set or TCGA set, we performed univariate Cox regression analysis to explore the association of patients' overall survival (OS) with microRNA expression. A total of 400 microRNAs significantly associated with OS (*P* < 0.05, Table S1) were discovered and displayed as grey or red dots in Figure 1A. Next, we further reduced the number of prognostic microRNAs available for risk model by random survival forests‐variable hunting (RSFVH) analysis, which were analysed by gradually removing one‐fourth of the least important genes at each step (Figure 1B). And we screened out 9 prognostic microRNAs based on importance scores (Figure 1C). Then, we brought the prognostic microRNAs into the risk prediction model formula and constructed 2^9^ − 1 = 511 combinations or possible signatures in the training dataset. Patients were assigned corresponding risk scores by each possible signature. The median risk score divided patients into high‐risk group or low‐risk group. ROC analyses were analysed, and AUC values were compared to finalize the best predictor from all the 511 signatures (Table S2). The final signature was screened out due to its maximum AUC value and best predictive performance (AUC signature = 0.69, Figure 1D), including five microRNAs (hsa‐miR‐10b, hsa‐miR‐148a, hsa‐miR‐155, hsa‐miR‐15b and hsa‐miR‐196b). And the risk model based on the expression values of above five microRNAs and their regression coefficients is as follows: Risk score = (0.18 × expression value of hsa‐miR‐10b) + (0.33 × expression value of hsa‐miR‐148a) + (0.59 × expression value of hsa‐miR‐155) + (0.68 × expression value of hsa‐miR‐15b) + (0.20 × expression value of hsa‐miR‐196b). The positive regression coefficients of these five microRNAs indicated that they were all risk genes and were associated with poor prognosis (Table 2).

**TABLE 2:** The microRNAs in the prognostic signature and their association with LGG prognosis in the training dataset (n = 525)

Database ID	Coefficient^a^	*P* value^a^	Gene expression level association with poor prognosis
hsa‐miR‐10b	0.18	<0.001	High
hsa‐miR‐148a	0.33	<0.001	High
hsa‐miR‐155	0.59	<0.001	High
hsa‐miR‐15b	0.68	<0.001	High
hsa‐miR‐196b	0.20	<0.001	High

^a^ Derived from the univariable Cox regression analysis in the training set.

### 3.2 The microRNA signature predicts survival of patients with diffuse lower‐grade glioma in the training and the independent dataset

**FIGURE 2:**
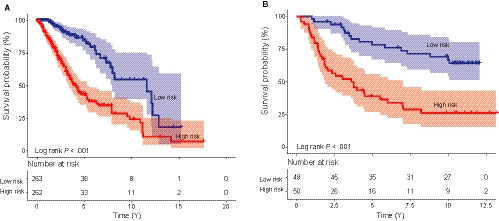
The prognostic microRNA signature predicts overall survival of patients with diffuse lower‐grade glioma. Kaplan‐Meier plots indicated patients could be classified into high‐ and low‐risk groups according to the signature in the training (A) and test (B) datasets, and P values were calculated by log‐rank test

When the median risk score was used as the cut‐off point, the five‐microRNA signature divided the training dataset into either the high‐risk (n = 262) or low‐risk group (n = 263). Kaplan‐Meier analysis identified the low‐risk group had significantly longer survival time than those in the high‐risk group (median survival: 11.59 years vs 4.08 years, *P* < 0.001; Figure 2A). Then, we treated the independent CGGA diffuse lower‐grade dataset with the specific follow‐up survival time and vital status (n* *= 99, Table 1) as the test dataset to validate the prognostic value of the five‐microRNA signature. We calculated the microRNA signature‐based risk scores of the patients and then separated patients into high‐ and low‐risk score group with the median risk score. Kaplan‐Meier verified that the five microRNA could distinguish the low‐risk group from the high‐risk group in the test dataset (log‐rank test *P* < 0.001; Figure 2B).

**FIGURE 3:**
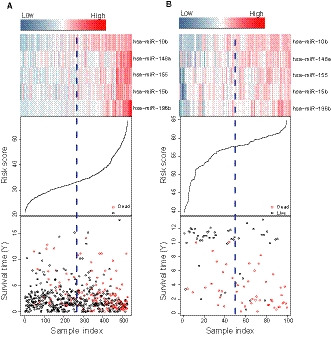
Risk score distribution, survival status and microRNAs expression patterns for patients in the training and test dataset

To further illustrate the relationship between gene expression of the five‐microRNA signature and survival, we showed each patient's gene expression, risk score and survival information in Figure 3. In the training dataset, the low expression of the five prognostic genes indicated the low the risk score and the long the patient's survival time. And patients with shorter survival times had higher risk scores and higher expression of five risk genes (Figure 3A). In the test dataset, patients with high five‐microRNAs expression have shorter life span and are more likely to die than patients with low five‐microRNAs expression (Figure 3B).

### 3.3 The five‐microRNA signature is an independent predictive factor

Through chi‐square test in the training or test dataset, we found microRNA signature was associated with clinical variables such as grade (Table 3).

**TABLE 3:** Association of the microRNA signature with clinical characteristics in LGG patients

Variables	Training set		*P*	Test set		*P*
	Low*	High*		Low*	High*	
Age						
>40	108	156	<0.001	16	25	0.07
≤40	155	106		34	23	
Gender						
Female	116	123	0.57	24	22	0.91
Male	147	139		26	27	
IDH mutation status						
Wild‐type	12	22	<0.001	12	18	0.21
Mutant	60	28		36	28	
Grade						
G2	161	94	<0.001	40	15	<0.001
G3	102	167		10	34	
Radio status						
NO	123	51	<0.001	3	6	0.48
YES	112	173		46	43	
Chemo status						
NO				26	18	0.13
YES				22	31	

*Low ≤ the median risk score. *High > the median risk score.

In addition, there are numerous known prognostic factors for LGG patients. Therefore, it is critical important to understand whether the microRNA signature could predict prognosis without relying on other factors. Multivariable Cox regression analysis showed that the microRNA signature could independently predict patients' clinical outcome in the training or test dataset (High‐risk group vs Low‐risk group, HRtraining = 2.91, 95% CI 1.92‐4.40, *P* < 0.001, n = 525; HRtest = 2.76, 95% CI 1.37‐5.55, *P* = 0.002, n = 99, Table 4). Besides, grade was also found to be a prognostic independent factor (G3 vs G2, HRtraining = 2.56, 95% CI 1.75‐3.75, *P* < 0.001, n = 525; HRtest = 2.84, 95% CI 1.45‐5.59, *P* = 0.005, n = 99, Table 4).

**TABLE 4:** Univariable and multivariable Cox regression analysis of the signature with LGG survival

Variables		Univariable analysis				Multivariable analysis			
		HR	95% CI of HR		*P*	HR	95% CI of HR		*P*
			lower	upper			lower	upper	
Training dataset (n = 525)									
Age	>40 vs ≤40	3.40	2.34	4.95	<0.001	2.54	1.75	3.68	<0.001
Sex	Male vs Female	1.14	0.81	1.60	0.46	1.20	0.85	1.70	0.30
IDH mutation status	Wild‐type vs Mutant	0.83	0.62	1.13	0.25	0.93	0.67	1.28	0.65
Grade	G3 vs G2	3.29	2.27	4.78	<0.001	2.56	1.75	3.75	<0.001
The microRNA signature	High risk vs low risk	3.77	2.51	5.65	<0.001	2.91	1.92	4.40	<0.001
Test dataset (n = 99)									
Age	>40 vs ≤40	1.91	1.09	3.33	0.02	1.57	0.87	2.83	0.13
Sex	Male vs Female	1.03	0.59	1.80	0.91	0.90	0.50	1.61	0.72
IDH mutation status	Wild‐type vs Mutant	1.58	0.87	2.88	0.14	1.19	0.64	2.21	0.58
Grade	G3 vs G2	4.60	2.54	8.35	<0.001	2.84	1.45	5.59	0.005
The microRNA signature	High risk vs low risk	3.70	2.03	6.74	<0.001	2.76	1.37	5.55	0.002

### 3.4 Comparison of IDH mutation and the five‐microRNA signature

**FIGURE 4:**
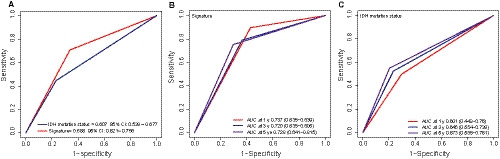
Comparison of the survival predictive power of the signature and IDH mutation by ROC (A). TimeROC analysis of the survival predictive power of the signature (B) and IDH mutation (C)

Considering the important role of IDH mutation in the prognosis prediction of LGG, we performed ROC analysis and compared the area under the ROC curve (AUC) of IDH mutation and the five‐microRNA signature to analyse their predictive power.^17^, ^18^ After congregating the TCGA and CGGA sets of data, we found a total of 216 patients with IDH mutation information. ROC analysis for vital status showed the predictive performance of the microRNA signature was better than IDH mutation status (AUCIDH = 0.607; AUCsignature = 0.688, Figure 4A). TimeROC analysis found the AUC of the signature was 0.737/0.720/0.728 at 1/3/5 years (Figure 4B), and the AUC of IDH was 0.601/0.646/0.673 at 1/3/5 years (Figure 4C) indicating that signature was better than that of IDH mutation in survival prediction.

### 3.5 Radiotherapy stratification analysis

**FIGURE 5:**
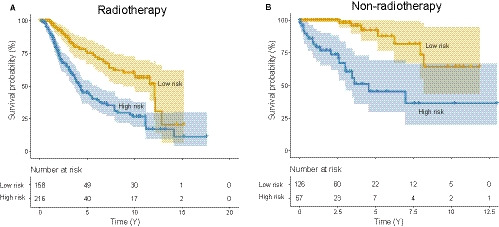
Radiotherapy stratification analysis. The five‐microRNA signature could further divide patients undergoing radiotherapy (A) or patients undergoing non‐radiotherapy (B) into two groups with significantly different survival

From the clinical information in Table1, we can find that most patients from the TCGA or CGGA datasets have clear radiotherapy information. To determine whether the signature can guide radiation therapy in LGG patients, we performed stratified analyses. As in the entire radiotherapy group, the overall survival time of the high‐risk (n = 216) patients was significantly shorter than that of low‐risk (n = 158) group patients (median survival 4.24 years vs 12.09 years, log‐rank test *P* < 0.001, Figure 5A). Patients undergoing non‐radiotherapy (n = 183) can also be divided into two groups with different prognosis by the signature (5‐year/10‐year survival rate: 45.60%/36.48% vs 92.40%/64.52%, log‐rank test *P* < 0.001, Figure 5B).

### 3.6 Function analysis of the five microRNAs

**FIGURE 6:**
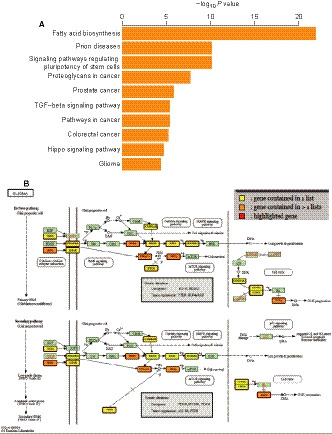
Functional enrichment analysis of the five microRNAs in the signature. (A) The top 10 differential pathways among the five microRNAs significantly enriched in multiple cancer‐related pathways. (B) The target genes of the five prognostic microRNAs involved in the Glioma pathway

To explore the role and function of microRNAs screened in this study, we first detected the target genes of the five microRNAs and then performed functional analysis of the target genes by mirPath (see method). Figure 6A showed the target genes of the five microRNAs were significantly enriched in multiple cancer‐related pathways, among which the glioma pathway was located in the top 10 differential pathways. Figure 6B displayed the target genes of the five prognostic microRNAs involved in the glioma pathway, indicating the pathway or mechanism in which the prognostic microRNAs might play a key role.

## 4 DISCUSSION

Since molecular features have integrated into glioma classification, the importance of molecular features in the prognosis of glioma is self‐evident. At present, IDH mutation and 1p/19g codeletion still have some defects in the prognosis evaluation and treatment guidance of patients with LGG. This study analysed 624 LGG patients and found a good indicator for prognosis prediction and radiotherapy guidance, which is named as a five‐microRNA signature. The signature could predict the survival of patients with LGG and determine whether patients can benefit from radiation therapy.

Compared with GBM patients with a median overall survival of 14‐17 months,^19^ patients with LGG are relatively benign with a median survival of 7 years.^20^ Although the new WHO glioma classification combined diffuse grade II with grade III gliomas into LGG, there are considerable differences among diffuse lower‐grade gliomas. From the view of tumour behaviour, some lower‐grade gliomas progress slowly, while others are as aggressive as glioblastoma. From the view of patients' outcome, some patients could survive 15 years and are sensitive to treatment whereas other patients have only 1 year of survival and no effective treatment.^21^ Thus, this article is devoted to finding novel molecular markers to assess the prognosis of each patient and determine personalized treatment. Based on microRNA expression profiles of 624 patients with LGG and multiple bioinformatics analysis methods, we find an expression‐based five‐microRNA signature that could categorize each patient as high risk or low risk in terms of survival. Two literatures on the prognostic microRNA signature of LGG can be found on Pubmed. Our five‐microRNA signature has several advantages over the two published microRNA signatures. First, our research cohort is the largest and contains over 600 patients with their expression data. Second, we evaluated the prognostic capabilities of IDH mutation and five‐microRNA signature, highlighting the advantages of our filtered signatures. Finally, we also discovered that our signature had the role of radiotherapy guidance, while the other two published signatures did not have this finding.

Isocitrate dehydrogenase (IDH)1 and IDH2 are key enzymes that catalyse the conversion of isocitrate to α‐ketoglutarate. IDH mutations cause a change in enzyme activity, resulting in a large amount of 2‐hydroxyglutarate (D2HG) being synthesized and accumulated. Since the discovery of IDH mutations in diffuse lower‐grade glioma,^22^ studies have confirmed IDH could be a prognostic biomarker for glioma.^23^, ^24^ Incorporating the IDH status into the new WHO classification highlights its prognostic ability. The five‐microRNA signature screened in this paper exceeded IDH mutation in prognostication through comparative analysis, illustrating its strong prognostic value. Moreover, in the new version of glioma classification, IDH NOS patients are difficult to assess prognosis. Therefore, the establishment of this microRNA signature can complement the prognosis of patients with IDH NOS glioma.

According to the European Association for Neuro‐Oncology (EANO) guideline, the treatment of patients with LGG is mainly conservative management and radiotherapy, and a few patients with high malignancy require combination chemotherapy. However, there is no consistent conclusion as to which patient can no longer continue to ‘watch and wait' but need radiotherapy or which patient have good radiotherapeutic response and can benefit from radiation therapy. Whether patients currently receive radiotherapy depends mainly on some prognostic indicators, such as age, Karnofsky performance score.^25^ This study found that the signature can classify patients after radiotherapy into high‐ and low‐risk groups with significant differences in prognosis, making the signature can be used to guide radiotherapy.

Notably, the five microRNAs in the signature are all risk factors. As for the functions of microRNAs in LGG, our GO and KEGG analysis found that the five risky microRNAs enriched in glioma related pathways. In the Pubmed database, we searched related studies to explore the role of the five microRNAs in glioma. Since the expression of miR‐10b was found to be up‐regulated and be closely related to the poor prognosis of glioma,^26^, ^27^, ^28^ researchers have discovered that miR‐10b may be involved in tumorigenesis by activating the p53 pathway^29^ and induced glioma cell invasion by miR‐10b/HOXD10/MMP‐14/uPAR signalling pathway.^30^ MiR‐10b down‐regulation inhabited proliferation, migration and invasion of glioma cell through regulating TGF‐β1 stimulation^31^ or homeobox B3 (HOXB3) expression.^32^ For miR‐148a, Wang et al^33^ the NF‐κB/miR‐148a/TGF‐β pathway was a critical mechanism of glioblastoma aggressiveness. Moreover, consistent with our study, researchers identified that miR‐148a was an oncogenic gene associated with glioma survival and affected EGFR activation or regulated glioma growth by mediating HIF1α and Notch signalling.^34^, ^35^ Encouragingly, circulating exosome miR‐148a was found in serum and promoted tumour progression by targeting CADM1 to activate STAT3 pathway, suggesting its clinical value.^36^ MiR‐155 has also been proven to be a circulating microRNA that is stable in plasma^37^ and could promote the proliferation and invasion of glioma. Yan et al^38^ believed that microRNA‐155 exerted oncogenic function through activating miR‐155/HBP1/Wnt/β‐catenin pathway. Other scientists reported that miR‐155 induced tumorigenesis was mediated by targeting γ‐aminobutyric acid A receptor 1 (GABRA1),^39^ caudal‐type homeobox 1 (CDX1)^40^ and F‐box and WD repeat domain containing 7 (FBXW7).^41^ Likewise, miR‐196b has also been suggested as an oncogene and could be used as a marker for the malignant progression of gliomas.^42^, ^43^ However, the specific mechanism of how miR‐196b promotes tumour progression has not yet been elucidated. MiR‐15b is the most controversial microRNA in the five‐microRNA signature. Several studies consider miR‐15b as an oncogene^44^ and a risk factor for glioma prognosis.^45^ Other studies, contrary to our finding, regarded miR‐15b as a tumour suppressor in the progression of glioma.^46^, ^47^, ^48^, ^49^ Therefore, the role of miR‐15b in glioma tumorigenesis remains unclear. Considering that the five microRNAs found in this study are all glioma prognostic risk genes, we still need to figure out whether there is some connection between the five microRNAs and how they work together to promote glioma progression.

In summary, our study findings revealed an expression‐based five‐microRNA signature can predict individual survival and radiotherapeutic response of patients with LGG. This paper highlights the promising potential of the novel five‐microRNA signature as a good prognostic biomarker for individual survival prediction and therapeutic decision making.

## CONFLICT OF INTEREST

The authors confirm that there are no conflicts of interest.

## AUTHORS' CONTRIBUTIONS

Zhang JH, Hou RQ, Pan YHL, Gao YH and Yang Y involved in data collection, data analysis, interpretation and drafting; Tian WQ and Zhu YB involved in study design, study supervision and final approval of the manuscript.

## ETHICAL APPROVAL

This article does not contain any studies with human participants or animals performed by any of the authors.

Informed consent: Informed consent was obtained from all individual participants included in the study. For specific informed consent, please refer to the source of public data.

## Supporting information

Table S1

Table S2

## Data Availability

Publicly available datasets were analysed in this study. This data can be found here: https://tcga.xenahubs.net/download/TCGA.LGG.sampleMap/microRNA_HiSeq_gene.gz,http://www.cgga.org.cn/download?file=download/20191128/CGGA.microRNA_array_198_gene_level.20191128.txt.zip&type=microRNA_array_198_gene_level&time=20191128.
